# Pharmacokinetics of Orally Administered Prednisolone in Alpacas

**DOI:** 10.3389/fvets.2021.745890

**Published:** 2021-10-22

**Authors:** Ricardo Videla, Carla Sommardahl, Joe Smith, Deanna M. W. Schaefer, Sherry Cox

**Affiliations:** ^1^Department of Large Animal Clinical Sciences, University of Tennessee, Knoxville, Knoxville, TN, United States; ^2^Department of Biomedical Sciences, Iowa State University, Ames, IA, United States; ^3^Department of Biomedical and Diagnostic Sciences, University of Tennessee, Knoxville, Knoxville, TN, United States

**Keywords:** prednisolone, pharmacokinetics, prednisone, alpaca, *Vicugna pacos*

## Abstract

This study aimed to determine the pharmacokinetics of prednisolone following intravenous and oral administration in healthy adult alpacas. Healthy adult alpacas were given prednisolone (IV, *n* = 4), as well as orally (PO, *n* = 6). Prednisolone was administered IV once (1 mg/kg). Oral administration was once daily for 5 days (2 mg/kg). Each treatment was separated by a minimum 4 month washout period. Samples were collected at 0 (pre-administration), 0.083, 0.167, 0.25, 0.5, 0.75, 1, 2, 4, 8, 12, and 24 h after IV administration, and at 0 (pre-administration), 0.25, 0.5, 0.75, 1, 2, 4, 8, 12, 24 after the first and 5^th^ PO administration. Samples were also taken for serial complete blood count and biochemistry analysis. Prednisolone concentration was determined by high pressure liquid chromatography. Non-compartmental pharmacokinetic parameters were then determined. After IV administration clearance was 347 mL/kg/hr, elimination half-life was 2.98 h, and area under the curve was 2,940 h^*^ng/mL. After initial and fifth oral administration elimination half-life was 5.27 and 5.39 h; maximum concentration was 74 and 68 ng/mL; time to maximum concentration was 2.67 and 2.33 h; and area under the curve was 713 and 660 hr^*^ng/mL. Oral bioavailability was determined to be 13.7%. Packed cell volume, hemoglobin, and red blood cell counts were significantly decreased 5 days after the first PO administration, and serum glucose was significantly elevated 5 days after the first PO administration. In conclusion, serum concentrations of prednisolone after IV and PO administration appear to be similar to other veterinary species. Future research will be needed to determine the pharmacodynamics of prednisolone in alpacas.

## Introduction

The growing population of South American camelids within the United States has resulted in the need for veterinary care of both common and uncommon disease processes in these species. There are currently no drugs approved by the Food and Drug Administration for camelids and pharmaceutical companies cannot economically justify seeking approval of drugs in these species. The pharmacokinetics for multiple classes of drugs for camelids have been described; including antibiotics (1–5), non-steroidal anti-inflammatories (6, 7), gastric acid suppressants (8–10), opioids (11, 12), and other pharmaceuticals (13, 14), however, no pharmacokinetic studies for prednisolone exist for camelids. Many of the dosage regimens used in camelids are empirical or extrapolated from species with different physiology and metabolism. However, several drugs have dissimilar kinetics in camelids as compared to other livestock species which can result in improper dosing or unwanted side effects. There is a need for understanding the pharmacokinetics of drugs in camelids to optimize medical treatment and reduce side effects.

Prednisone and prednisolone are synthetic analogs of cortisol. Prednisone is more affordable than prednisolone but it needs to be converted by the liver to its active metabolite, prednisolone, to have a therapeutic effect. Prednisone is readily converted into prednisolone in humans and dogs (15, 16). However, in the cat and horse there is evidence that prednisone is not efficiently metabolized to prednisolone, and therefore not therapeutic (17, 18). There are no published studies to date to determine if camelids are able to convert prednisone into prednisolone. Prednisolone is available in oral and injectable formulations for use in some domestic species. In camelids, oral administration would be preferred as venous access can be challenging for most owners to administer and stressful to the animal.

Even though steroids are fundamental for the treatment of certain conditions such as autoimmune diseases, there can be adverse side effects which often make practitioners wary of using this therapy. Exogenous steroids can cause suppression of the hypothalamo-pituitary-adrenal (HPA) axis, which often leads to harmful side effects if discontinued abruptly, especially after prolonged therapy. Other side effects include polyuria/polydipsia, changes in appetite, muscle atrophy, susceptibility to infection, gastrointestinal ulceration, changes in liver function, and abortion (19). Given the prominent adverse side effects of glucocorticoid therapy, it is important to use the lowest effective dose possible. Determining the pharmacokinetics of oral prednisolone is an essential step in determining the most appropriate dose for camelids.

To date there are no studies analyzing the pharmacokinetics of orally administered corticosteroids in camelids. The aim of this study is to determine the bioavailability and pharmacokinetics of prednisolone in alpacas after oral administration, and to evaluate possible side effects during and after a 5 day treatment. We hypothesize that oral administration of prednisolone will result in blood levels comparable to levels of clinical value in other species and that a 5 day course of treatment will result in no or minimal side effects.

## Materials and Methods

This study was approved by the Institutional Animal Care and Use Committee of the University of Tennessee (protocol #2400–1215). Four clinically healthy alpacas were used, housed in box stalls at least 24 h before and during the experiment. Alpaca ages and weights (mean ± standard deviation) were 8.0 ± 4.3 years and 68.8 ± 9.7 kgs. Two intravenous jugular catheters were placed (one in each jugular vein) the day prior to the intravenous component of the study. Each alpaca (*n* = 4) was administered 1.0 mg/kg prednisolone (Prednisolone, USP, Rockville, MD, USA), intravenously in one catheter and samples were collected from the other catheter. Blood samples were collected at 0 (pre-administration), 5, 10, 15, 30, and 45 min as well as 1, 2, 4, 8, 12, and 24 h after administration and were centrifuged at 1,000 g for 15 min. Plasma was removed and stored at −80°C until analysis.

After a washout period of 4 months six alpacas were housed in box stalls and had intravenous catheters placed in the jugular vein. Prednisolone tablets (PrednisTab^®^, Lloyd Inc., Shenandoah, IA), were dosed at 2 mg/kg every 24 h for 5 days to six alpacas. Blood samples were collected at 0 (pre-administration), 15, 30, and 45 min as well as 1, 2, 4, 8, 12, 24 (pre-second administration) hours. On days 2–4 blood samples were collected at peak (2 h after administration) and trough times (immediately prior to drug administration). On the 5^th^ day samples were collected at 0 (pre-administration of the fifth dose), 15, 30, and 45 min as well as 1, 2, 4, 8, and 12 h after the last administration. Samples were immediately centrifuged after collection with the serum stored at −80°C until analysis. Additionally, before the initial drug administration (day 1), on the day of the last dose (day 5), and 5 days after the last dose (day 10), whole blood was collected into a tube containing ethylenediaminetetraacetic acid (EDTA) anticoagulant for complete blood count (CBC) and a tube containing heparin anticoagulant for plasma chemistry testing. These samples were submitted to the University of Tennessee Veterinary Medical Center (UTCVM) clinical pathology laboratory, with testing performed according to the laboratory standard operating procedure using an ADVIA 2,120 hematology analyzer (Siemens, Munich, Germany) and a Cobas C501 chemistry analyzer (Roche Diagnostics, Basel,Switzerland) (20).

Analysis of prednisolone in plasma samples was conducted using reversed phase HPLC. The system consisted of a 2695 separations module and a 2487 UV detector (Waters, Milford, MA, USA.). Separation was attained on a Waters Symmetry Shield RP_18_ 4.6 x 150 mm (5 μm) protected by a 5 μm Symmetry Shield RP_18_ guard column. The mobile phase was an isocratic mixture of 100 mM ammonium acetate pH 4.0 with concentrated glacial acetic acid and acetonitrile (70:30). It was prepared fresh daily using double-distilled, deionized water filtered (0.22 μm) and degassed before use. The flow rate was 1.0 ml/min and UV absorbance was measured at 254 nm.

Prednisolone was extracted from plasma samples using liquid-liquid extraction. Briefly, previously frozen plasma samples were thawed and vortexed, and 500 μL was transferred to a clean screw-top test tube followed by 25 μL internal standard (10 μg/mL methylprednisolone). Methylene chloride (3 mL) was added and the tubes were rocked for 20 min and then centrifuged for 20 min at 1,000 X *g*. The organic layer was transferred to a clean tube and evaporated to dryness with nitrogen gas. Samples were reconstituted in 250 μL of mobile phase and 100 μL was analyzed.

Standard curves for plasma analysis were prepared by fortifying untreated, pooled alpaca plasma with prednisolone to produce a linear concentration range of 5–1,000 ng/mL. Calibration samples were prepared exactly as plasma samples. Average recovery for prednisolone was 96% while intra and inter-assay variability ranged from 2.5 to 5.8% and 1.01 to 6.25%, respectively. The lower limit of quantification was 5 ng/mL and the limit of detection was 2.5 ng/mL.

### Pharmacokinetic Analysis

Pharmacokinetic parameters for prednisolone were calculated using Phoenix WinNonlin 6.4 (Certara USA, Inc., Princeton, New Jersey 08540, USA). Values for elimination rate constant (λ_z_), plasma half-life (t_½_), plasma concentration back extrapolated to time 0 (C_0_), maximum plasma concentration (C_max_), time to maximum plasma concentration (T_max_), total body clearance (Cl), volume of distribution (Vd_area_), apparent volume of distribution at steady-state (Vd_ss_), mean residence time (MRT_0−∞_), area under the plasma concentration time curve from time 0 to infinity (AUC_0−∞_) and area under the plasma concentration time curve from time 0 to last time point (AUC_0−Last_) were calculated from non-compartmental analysis. The AUC was calculated using the log-linear trapezoidal rule. Variability in pharmacokinetic parameters was expressed as the standard deviation. In the case of the half-life, harmonic mean and pseudostandard deviation were used. Absolute systemic bioavailability (F) of prednisolone was calculated from noncompartmental parameters with the following equation:


$$\begin{array}{l} \text{F}=\frac{{\text{AUC}}_{0-\infty \left(\text{PO}\right)}x\;{\text{Dose}}_{\left(\text{IV}\right)}}{{\text{AUC}}_{0-\text{∞}\left(\text{IV}\right)}x\;{\text{Dose}}_{\left(\text{PO}\right)}} \end{array}$$

The global extraction ratio (E_body_) was calculated as reported by Toutain and Bousquet-Melou (21), with:


$$\begin{array}{l} {\text{E}}_{\text{body}}=\text{Systemic clearance}/\text{Cardiac output} \end{array}$$

First calculated for each individual animal, and then combined for a mean value as previously described (22). With the alpaca cardiac output calculated as follows:


$$\begin{array}{l} \text{Cardiac output}=180\times \text{BW}{\left(\text{kg}\right)}^{-0.19} \end{array}$$

### Statistical Analysis

Pharmacokinetic variables for prednisolone following oral and intravenous administration were calculated with a commercial computer software program (Phoenix 6.30, Pharsight Corp.). Pharmacokinetic parameters were tested for normality of distribution and equal variance (Graphpad Prism, La Jolla, CA) when data were normally distributed and had equal variances; a *t* test was performed to determine whether differences existed between pharmacokinetic parameters from the IV prednisolone administration to Day 1 of oral administration, as well as Day 1 and Day 5 oral administration of prednisolone. Values of *P* < 0.05 were considered significant for all statistical tests.

CBC and plasma chemistry results were inspected for abnormalities using alpaca reference intervals established in the UTVMC clinical pathology laboratory. Additionally, paired *t*-tests were used to evaluate for statistical difference between values on days 0, 5, and 10 (MedCalc Software Ltd, version 20.009).

## Results

**Figure 1:**
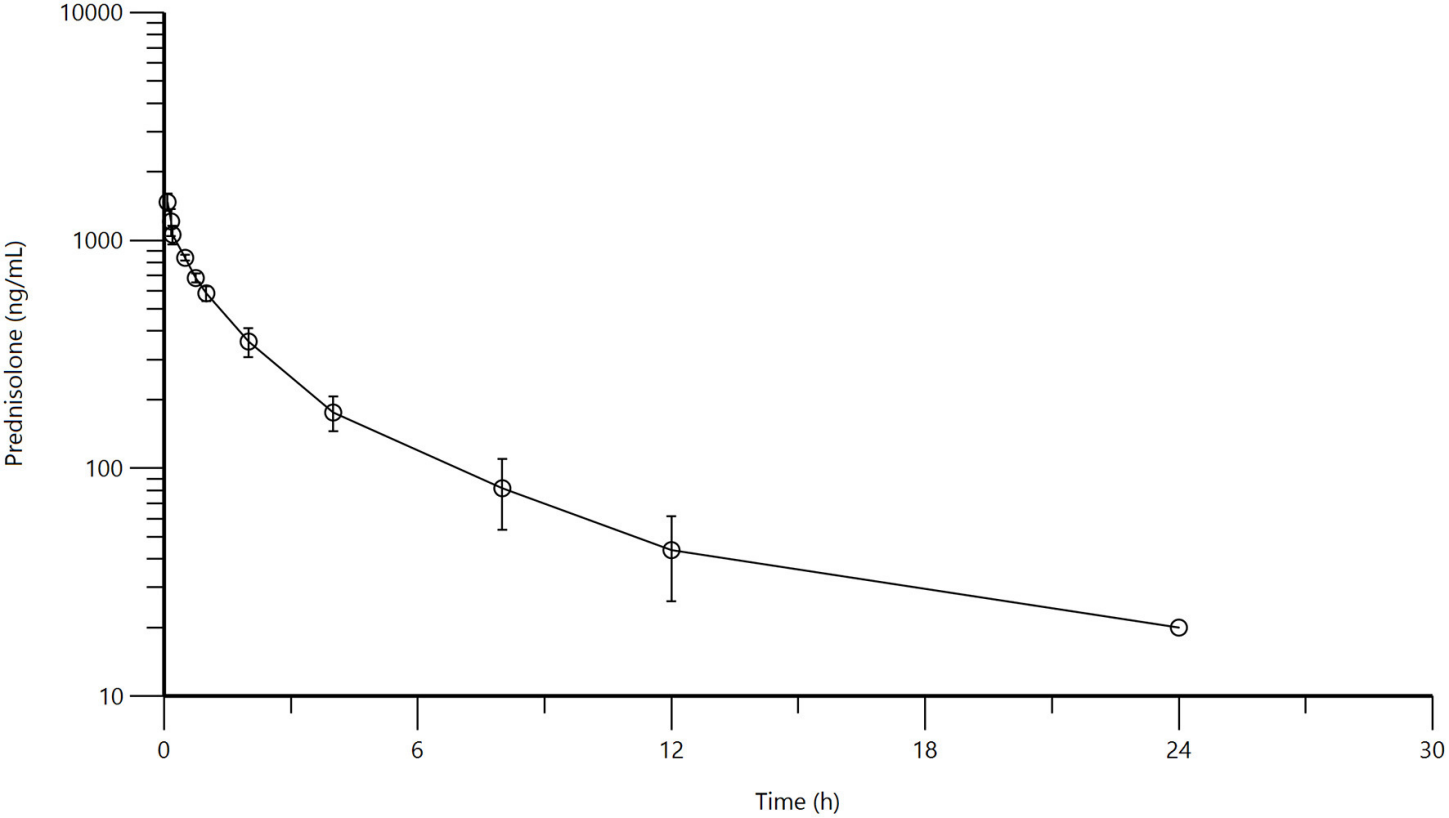
Mean plasma prednisolone concentration (logarithmic scale) vs. time (h) profiles for adult alpacas (*n* = 4) following intravenous (IV) single dose administration of 1.0 mg/kg of prednisolone. Mean is represented by an open circle with error bars representing standard deviation.

**Figure 2:**
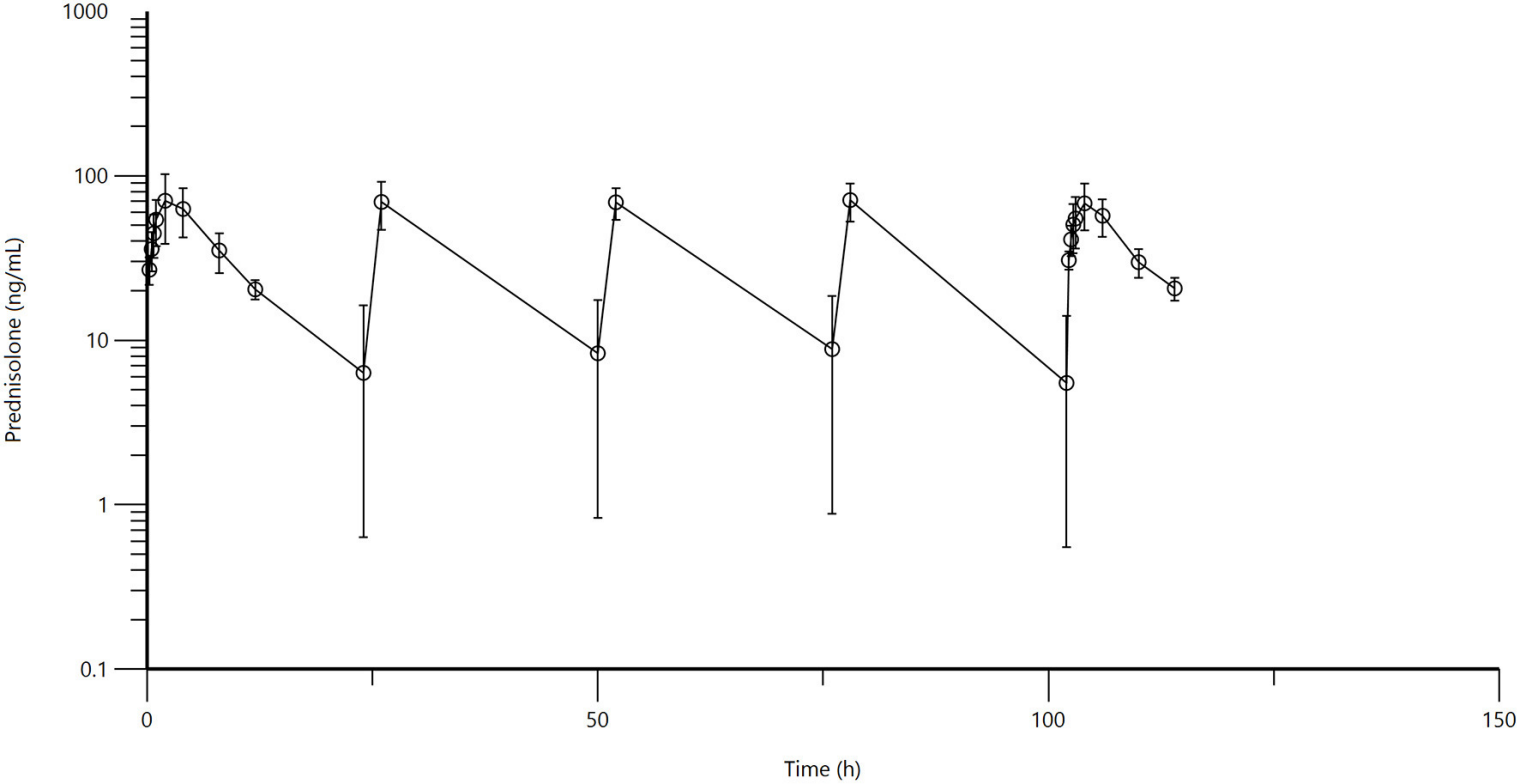
Figure 1: Mean plasma prednisolone concentration (logarithmic scale) vs. time (hr) profiles for adult alpacas (*n* = 6) following oral (PO) muliple dose administration of 2.0 mg/kg of prednisolone. Mean is represented by an open circle with error bars representing standard deviation. Doses were administered at 0, 24, 48, 72, and 96 h.

Mean and standard deviation for plasma prednisolone concentrations for the single IV (Figure 1) and multidose PO study (Figure 2) are represented graphically. Pharmacokinetic parameters were summarized in Table 1. The mean of trough concentrations was similar on each of the days (D2, 18 ng/mL; D3, 17 ng/mL; and D4, 18 ng/mL).

**Table 1:** Pharmacokinetic parameters (mean ± SD) in alpacas on Day 1 and Day 5 after multi-dose oral administration of 2 mg/kg prednisolone (*n* = 6) and IV administration of 1 mg/kg (*n* = 4).

**Pharmacokinetic parameter**	**IV Administration**	**PO Administration mean ± SD**	**PO Administration day 5**
		**mean ± SD**	**mean ± SD**
		**day 1**	**day 5**
Terminal half-life^*^ (h)	2.98 ± 0.795	5.27 ± 1.73	5.39 ± 1.36
Elimination rate constant, λ_z_ (1/h)	0.232 ± 0.062	0.131 ± 0.043	0.128 ± 0.032
C_0_ (ng/mL)	1,795 ± 129	NA	NA
T_max_ (h)	NA	2.67 ± 1.03	2.33 ± 0.82
C_max_ (ng/mL)	NA	74 ± 31	68 ± 21
Cl (mL/h/kg)	347 ± 54	NA	NA
V_dss_ (mL/kg)	1,295 ± 242	NA	NA
V_d(area)_ (mL/kg)	1,554 ± 372	NA	NA
AUC_0−∞_ (h·ng/mL)	2,940 ± 474	713 ± 140	660 ± 101
AUC_0−Last_ (h·ng/mL)	2,797 ± 480	538 ± 155	499 ± 111
AUC_extrapolated_ (%)	6.0 ± 4.6	26.5 ± 11.5	25.7 ± 8.6
MRT_0−∞_(h)	3.86 ± 1.17	9.22 ± 3.17	8.73 ± 1.97
F (%)	NA	13.7 ± 3.5	NA
Extraction (%)	4.16 ± 0.58	NA	NA

* *Harmonic mean; Elimination rate constant (λ_z_), terminal half-life $\left({\text{t}}_{1/2}\right)$, plasma concentration back extrapolated to time 0 (C_0_), maximum plasma concentration (C_max_), time to maximum plasma concentration (T_max_), area under the plasma concentration time curve from time 0 to infinity (AUC_0−∞_), area under the plasma concentration time curve from time 0 to last time point (AUC_0−Last_), mean residence time (MRT_0−∞_), Vd_area_, volume of distribution; Vd_ss_, apparent volume of distribution at steady-state, Cl, total body clearance; F, absolute systemic bioavailability*.

A non-compartmental model was used to evaluate plasma concentrations after both IV and PO dosing. The half-life, volume of distribution at steady-state and clearance for prednisolone after IV administration were 2.98 ± 0.795 h, 1,295 ± 242 mL/kg, 347 ± 54 mL/h/kg, respectively. The half-life of prednisolone after oral dosing once a day for 5 days was 5.39 ± 1.36 h. The mean prednisolone bioavailability after oral dosing was 13.7%. The extrapolated area under the curve was 6.0 ± 4.6% for intravenous administration; 26.5 ± 11.5% (after the first oral administration) and 25.7 ± 8.6% (after last oral administration). Observed extraction ratio was 4.16 ± 0.58.

When IV vs. PO administration was statistically compared, significant differences were observed for lambda Z (*p* = 0.0153), area under the curve last (*p* = <0.0001), mean residence time (*p* = 0.0129), and extrapolated area under the curve (*p* = 0.0135). The *P* value for comparison of elimination half-life was low (*p* = 0.0610), but not significantly different. There were no significant differences between any of the pharmacokinetic parameters between Day 1 and Day 5 for oral administration.

**Figure 3:**
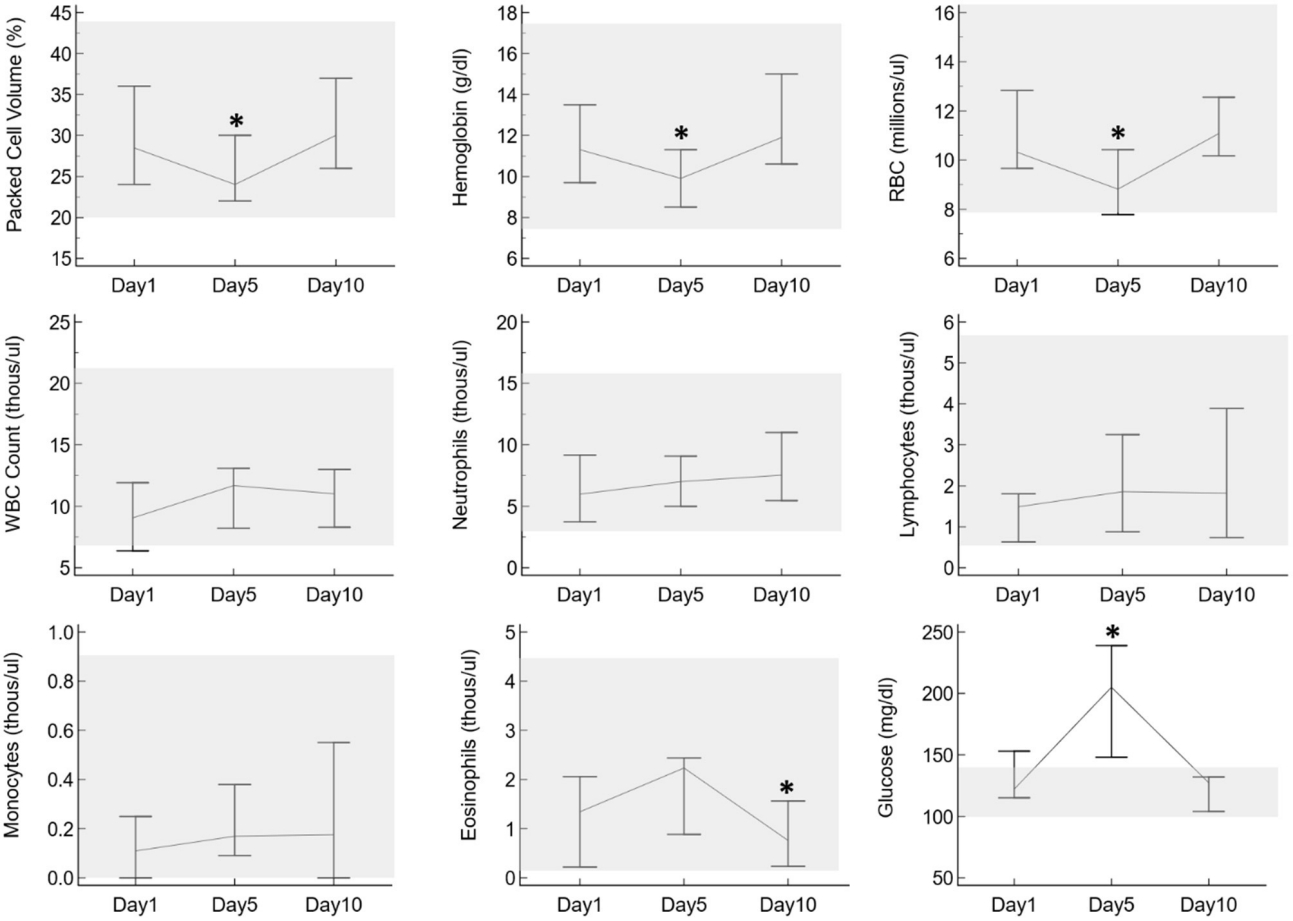
Selected CBC and plasma biochemistry results from six alpacas before oral prednisolone (day 1), after 5 days of oral prednisolone (day 5), and after a 5-day post-administration washout (day 10). The vertical lines indicate the range of results for each day, with lines connecting at the medians. The gray shaded boxes highlight the reference interval for each parameter as established for alpacas in the UTVMC clinical pathology laboratory. Asterisks indicate days in which results are significantly different from the other two days.

No clinical adverse effects were observed in any of the alpacas at any point during the study period. On the CBC, packed cell volume (PCV), hemoglobin, and red blood cell counts were significantly lower on day 5 compared to days 0 and 10, and eosinophil numbers were significantly lower on day 10 compared to days 0 and 5, but all results were still within the reference intervals with the exception of one alpaca with a very mildly decreased red blood cell count on day 5 (Figure 3). Additionally, glucose was significantly higher on day 5 compared to days 0 and 10 and was above the reference interval in all 6 alpacas on that day. There were trends toward increasing white blood cell count, neutrophils, lymphocytes, and monocytes over time, but these were not statistically significant and did not go above the reference interval in any individuals. No other relevant changes were identified on CBC or chemistry results.

## Discussion

To the author's knowledge, this is the first study to determine the pharmacokinetics of prednisolone in alpacas. Glucocorticoids, such as prednisolone, are a powerful and effective therapeutic tool for many inflammatory disease processes. This class of drugs includes dexamethasone, prednisone, prednisolone, and hydrocortisone among others. They are used as anti-inflammatory agents, immunosuppressives, for the treatment of lymphoma (cytotoxicity toward neoplastic lymphocytes), or to replace glucocorticoid activity in patients with adrenal insufficiency. They can be clinically beneficial in diseases where inflammation has detrimental effects such as uveitis, immune mediated diseases, asthma, inflammatory bowel disease, skin allergies, certain neoplasias, lameness, and many neurologic conditions (16, 19). Descriptions of the use of prednisolone in the camelid medical literature are sparse. Historical reports describe prednisolone for chemotherapy of lymphosarcoma or lymphoma (23, 24). Anti-inflammatory doses are used for management of soft tissue injury from causes such as cerebrospinal nematodiasis and rattlesnake envenomation (25–27). Additional descriptions exist for the treatment of certain dermatological conditions (28). Topical use is described in the species for ophthalmologic cases (29).

There are limited reports of pharmacokinetic data for prednisolone among large animal species. Studies exist for cattle, horses, and sheep (30–32). The clearance observed in the alpacas in this study (0.347 L/kg/hr) is similar to the ranges observed in horses (0.235–0.374), as well as a cattle (0.42) and less than those reported for sheep (0.93) (30, 31, 33). The elimination half-life of intravenous prednisolone reported in the alpacas in this study (2.98 h) is similar to the elimination half-life reported for cattle (3.6), but longer than horses (1.15–1.65) or sheep (0.85) after intravenous administration (30–33). Table 2 displays pharmacokinetic information for prednisolone in sheep, cattle, horses as well as the alpacas from this study. The bioavailability demonstrated by the alpacas in our study after oral administration was low (13.7%). Reports of the oral bioavailability of prednisolone in large animal species are limited, however, this is similar to the low range of bioavailability (18%) reported for prednisolone tablets in dogs (34). When comparing the pharmacokinetic parameters from our study to other large animal studies, it is important to note the limit of quantification. When limits of quantification are more sensitive, there is the potential for some pharmacokinetic parameters, such as elimination half-life, to be increased (22). All of the large animal assays employed similar sensitivity (2–3 ng/mL), so it is likely that the pharmacokinetic parameter differences are true species differences instead of analytical method discrepancies.

**Table 2:** Comparative pharmacokinetic parameters for prednisolone in other large animal species.

**Species**	**Dose**	**Product**	**Route**	**T_**1/2**_ (λz) hr**	**Cl l/kg/hr**	**LLOQ**	**Reference**
Cattle	600 μg/kg	Prednisolone 21-Sodium Succinate	IV	3.6 ± 1.177	0.42 ± 0.05	2 ng/mL	Toutain et al. (31)
Sheep	1 mg/kg	Prednisolone	IV	0.85 ± 0.14	0.93 ± 0.13	2.0 ng/mL	Alvinerie et al. (32)
Horses	0.6 mg/kg	Prednisolone 21-Sodium Succinate	IV	1.65 ± 0.292	0.235 ± 0.039	2–3 ng/mL	Toutain et al. (30)
Horses	450 mg (total)	Prednisolone 21-Sodium Succinate	IV	1.15 ± 0.233	0.374 +/- 0.047	N/A	Chen et al. (33)
Alpacas	1 mg/kg	Prednisolone Sodium Succinate	IV	2.98 ± 0.795	0.347 ± 0.054	2 ng/mL	Present study

Due to the multiple downstream effects of prednisolone, there currently are not many recommendations regarding therapeutic concentrations of prednisolone in the veterinary literature. In beagles administered oral prednisolone at 2.0 mg/kg, maximum plasma concentrations of 58.2 ng/mL have been observed, this is similar to the maximum concentrations of 74 and 68 ng/mL observed in the alpacas in this study (35). This dosage in dogs has been described for the use as an anti-inflammatory agent as well as and antineoplastic agent (19, 36). While more investigation is necessary, this comparative observation may suggest that the oral dosing regimen used in the alpacas in this study may have similar plasma concentrations to other species.

Adverse effects reported in association with the administration of steroids in veterinary medicine include: leukocytosis with neutrophilia, monocytosis, lymphopenia, and eosinopenia. Also a mild elevation in albumin and in liver enzymes has been reported after treatment with steroids in dogs (37). In this alpaca study, there was a significantly decreased PCV, hemoglobin, and red blood cell count after 5 days of prednisolone administration, which was resolved at the recheck 5 days later. In the majority of individuals, all three of these parameters were still within the reference interval at all time points, so this may not be a clinically relevant change. Nevertheless, based on these results it may be worthwhile to periodically monitor for anemia in alpacas that are treated with prednisolone. There was also a mild but significant decrease in eosinophils noted at day 10. Eosinopenia can be a consequence of corticosteroids, but is unlikely to be clinically relevant. When serum biochemistry information was evaluated, the only consistent change across all animals was mild hyperglycemia noted 5 days after administration of prednisolone, which may be due to the gluconeogenic effects of corticosteroids.

Future directions for prednisonolone in alpacas include pharmacodynamic studies. One key area for further pharmacodynamic investigation is the use of prednisolone for anti-neoplastic therapy, considering the alpaca's role as a primarily companion animal, and the propensity of older alpacas to develop cancer (24, 38, 39). Additional research is needed to elucidate the effects that body condition could have on the pharmacokinetics of prednisolone in alpacas, as overconditioning (increased body fat percentage) in some species, such as cats, is associated with higher serum concentrations (40). Another additional consideration is the effect of multiple drug administration on the pharmacokinetics of prednisolone in camelids. In dogs, the downregulation of P-glycoprotein *via* the administration of ketoconazole lead to an increased area under the curve for prednisolone due to the ketoconazole-induced P-glycoprotein inhibition (41). With the potential for biochemical and hematopoietic adverse effects, non-linear mixed-effect modeling could be utilized to investigate the potential factors for these adverse effects when prednisolone is administered to alpacas (42). Limitations of this study include the small sample size, however, in veterinary pharmacology studies sample sizes of 4–6 animals are typically customary for describing pharmacokinetic parameters (43).

In conclusion, prednisolone administered at one IV dose of 1 mg/kg or 5 consecutive oral daily doses of 2 mg/kg was well-tolerated by alpacas in this study. Intravenous pharmacokinetics had similarities to cattle, specifically elimination half-life and plasma clearance. Evaluation of complete blood counts and serum biochemistry data suggested mild hyperglycemia and neutrophilia may be encountered from prednisolone administration. The concentrations reached by repeated oral administration are similar to those noted in other veterinary species when dosed at similar regimens. Future studies will be necessary to evaluate the pharmacodynamics of prednisolone when evaluating the effects of prednisolone administration to alpacas.

## Data Availability Statement

The original contributions presented in the study are included in the article/supplementary material, further inquiries can be directed to the corresponding author/s.

## Ethics Statement

The animal study was reviewed and approved by Institutional Animal Care And Use Committee.

## Author Contributions

RV, CS, DS, and SC developed the study design. RV and CS contributed to sample collection. SC developed the analytical method. SC and JS contributed to pharmacokinetic analysis. RV, CS, DS, JS, and SC all contributed to data interpretation and analysis. All authors contributed to manuscript construction.

## Funding

This work was entirely funded by the Alpaca Research Foundation.

## Conflict of Interest

The authors declare that the research was conducted in the absence of any commercial or financial relationships that could be construed as a potential conflict of interest.

## Publisher's Note

All claims expressed in this article are solely those of the authors and do not necessarily represent those of their affiliated organizations, or those of the publisher, the editors and the reviewers. Any product that may be evaluated in this article, or claim that may be made by its manufacturer, is not guaranteed or endorsed by the publisher.
